# Supplementary material for: Content-rich biological network constructed by mining PubMed abstracts
Source: BMC Bioinformatics. 2004 Oct 8;5:147. doi: 10.1186/1471-2105-5-147 (PMC528731; doi:10.1186/1471-2105-5-147)
Supplement: Additional File 2 — The original results of the above study (non-essential files are deleted to keep the file size under the limit set by BMC bioinformatics). [file 1471-2105-5-147-S2.bz2 › chilibotAdditionalFile2/dip50/196ID8652557E679/html/ERCC3_ERCC2.html]

 


 **ERCC3** and **ERCC2** 
  
Found 122 abstracts in PubMed, retrieved 50.  
 

 What does Google say? 
 PDF only 
| .edu only 

---

**Interactive relationship** (e.g. stimulation, inhibition, etc)

**Neutral relationship**- This interaction involves 1 the integrity of helix 12 of the LBD AF2 and 2 p62 and XPD  [ **ERCC2** ] , two subunits of the core TFIIH  [ **ERCC3** ] .  Ref: 10949034 Mol Cell, 2000
- The xeroderma pigmentosum group D XPD  [ **ERCC2** ]  protein is a subunit of transcription factor TFIIH  [ **ERCC3** ]  with DNA helicase activity.  Ref: 11709541 Hum Mol Genet, 2001
- XPD  [ **ERCC2** ]  is a component of the TFIIH  [ **ERCC3** ]  transcription factor, which is essential for RNA polymerase II elongation.  Ref: 11334141 Antisense Nucleic Acid Drug Dev, 2001
- Cloning of a human homolog of the yeast nucleotide excision repair gene MMS19 and interaction with transcription repair factor TFIIH  [ **ERCC3** ]  via the XPB  [ **ERCC3** ]  and XPD  [ **ERCC2** ]  helicases.  Ref: 11071939 Nucleic Acids Res, 2000
- As biochemists, we have characterized each component of TFIIH  [ **ERCC3** ] , three of which are XPB  [ **ERCC3** ]  and XPD  [ **ERCC2** ]  helicases and cdk7, a cyclin dependent kinase.  Ref: 11412842 FEBS Lett, 2001
- one of the roles of XPD  [ **ERCC2** ]  helicase is to anchor the cdk7 kinase to the core TFIIH  [ **ERCC3** ] .  Ref: 11412842 FEBS Lett, 2001
- Using immunoprecipitates from cells coinfected with baculoviruses, we further validated the bridging function of XPD  [ **ERCC2** ] , which anchors CAK to the core TFIIH  [ **ERCC3** ] .  Ref: 11445587 J Biol Chem, 2001
- Contrary to gene expression data, XPB  [ **ERCC3** ]  protein was found at high levels and XPD  [ **ERCC2** ]  at low levels in GDs 10 12 embryos and yolk sacs.  Ref: 11425516 Mutat Res, 2001
- The two helicases, XPB  [ **ERCC3** ]  and XPD  [ **ERCC2** ] , are components of the basal transcription factor TFIIH  [ **ERCC3** ] , which has a dual role in NER and initiation of transcription.  Ref: 11037299 Adv Genet, 2001
- The XPD  [ **ERCC2** ]  gene encodes a subunit of the transcription factor II H TFIIH  [ **ERCC3** ] , a complex involved in nucleotide excision repair NER and basal transcription.  Ref: 11737070 Clin Exp Immunol, 2001
- Taken together, our results show that the p52 Tfb2 subunit of TFIIH  [ **ERCC3** ]  regulates the function of XPB  [ **ERCC3** ]  through pair wise interactions as described previously for p44 and XPD  [ **ERCC2** ] .  Ref: 12080057 J Biol Chem, 2002
- p44 SSL1, the regulatory subunit of the XPD  [ **ERCC2** ]  RAD3 helicase, plays a crucial role in the transcriptional activity of TFIIH  [ **ERCC3** ] .  Ref: 10924514 J Biol Chem, 2000
- Trichothiodystrophy TTD is a rare hereditary multisystem disorder associated with defects in nucleotide excision repair NER as a consequence of mutations in XPD  [ **ERCC2** ] , XPB  [ **ERCC3** ]  or TTDA, three genes that are all related to TFIIH  [ **ERCC3** ] , the multiprotein complex involved in NER and transcription.  Ref: 12393803 Hum Mol Genet, 2002
- In both processes, TFIIH  [ **ERCC3** ]  is implicated with local DNA unwinding, which is attributed to its helicase subunits XPB  [ **ERCC3** ]  and XPD  [ **ERCC2** ] .  Ref: 11141066 Biochemistry, 2001
- We also show that mutations in CSB, as well as in XPB  [ **ERCC3** ]  and XPD  [ **ERCC2** ]  genes, all of which confer CS, disturb the RNA pol I TFIIH  [ **ERCC3** ]  interaction within the CSB IP 150.  Ref: 12419226 Mol Cell, 2002
- The p44 subunit plays a crucial role in the overall activity of the transcription DNA repair factor TFIIH  [ **ERCC3** ]  on the one hand its N terminal domain interacts with and regulates the XPD  [ **ERCC2** ]  helicase,.  Ref: 11319235 J Biol Chem, 2001
- S. cerevisiae RAD25  [ **ERCC3** ]  SSL2, RAD3, RAD26, YKU80 HDF2 and RAD54 are the homologue for XPB  [ **ERCC3** ]   **ERCC3** , XPD  [ **ERCC2** ]   **ERCC2** , CSB ERCC6, Ku80 XRCC5 and ATR X HX2, respectively.  Ref: 12638184 Kokuritsu Iyakuhin Shokuhin Eisei Kenkyusho Hokoku, 2002
- These findings suggest that the nuclear entry of XPD  [ **ERCC2** ]  depends on its complexation with other proteins in the cytoplasm, possibly other components of the TFIIH  [ **ERCC3** ]  complex.  Ref: 11266560 Nucleic Acids Res, 2001
- TFIIH  [ **ERCC3** ]  is a multifunctional RNA polymerase II general initiation factor that includes two DNA helicases encoded by the Xeroderma pigmentosum complementation group B XPB  [ **ERCC3** ]  and D XPD  [ **ERCC2** ]  genes and a cyclin dependent protein kinase encoded by the CDK7 gene.  Ref: 11331764 Proc Natl Acad Sci U S A, 2001
- General transcription factor IIH TFIIH  [ **ERCC3** ]  consists of nine subunits cyclin dependent kinase 7 Cdk7, cyclin H and MAT1 forming the Cdk activating kinase or CAK complex, the two helicases Xpb  [ **ERCC3** ]  Hay and Xpd  [ **ERCC2** ] , and p34, p44, p52 and p62 refs 1 3.  Ref: 12853965 Nature, 2003
- XPD  [ **ERCC2** ]  mutations prevent TFIIH  [ **ERCC3** ]  dependent transactivation by nuclear receptors and phosphorylation of RARalpha.  Ref: 11955452 Cell, 2002
- Moreover, when XPD  [ **ERCC2** ]  mutations prevent interaction with the p44 subunit of TFIIH  [ **ERCC3** ] , transactivation directed by certain nuclear receptors is inhibited, regardless of TTD versus XP phenotype, thus explaining the overlapping symptoms.  Ref: 12820975 Mol Cell, 2003

**Non-interactive relationship** (e.g. studied together, co-existance, homology, etc.)

- The repair deficient form of trichothiodystrophy TTD most often results from mutations in the genes XPB  [ **ERCC3** ]  or XPD  [ **ERCC2** ] , encoding helicases of the transcription repair factor TFIIH  [ **ERCC3** ] .  Ref: 11062469 Nat Genet, 2000
- Transcriptional regulation of the TFIIH  [ **ERCC3** ]  transcription repair components XPB  [ **ERCC3** ]  and XPD  [ **ERCC2** ]  by the hepatitis B virus x protein in liver cells and transgenic liver tissue.  Ref: 11278765 J Biol Chem, 2001
- Cell lines defective in other genes involved in NER XPB  [ **ERCC3** ] , XPD  [ **ERCC2** ] , and XPG, together with a mutant defective in nonhomologous end joining XRCC5, showed only mild sensitivity.  Ref: 11027268 Mol Cell Biol, 2000
- In UV sensitive TTD, the TFIIH  [ **ERCC3** ]  transcription factor containing XPB  [ **ERCC3** ]  and XPD  [ **ERCC2** ]  helicase activities necessary for both transcription initiation and DNA repair is damaged.  Ref: 11369901 J Am Acad Dermatol, 2001
- In contrast to cells bearing mutations in other components of the nucleotide excision repair apparatus XPB  [ **ERCC3** ] , XPD  [ **ERCC2** ] , XPG and CSB, cells defective for the ERCC1 XPF structure specific nuclease are highly sensitive to cisplatin.  Ref: 12202770 Nucleic Acids Res, 2002
- Moreover, this study shows how the activity of the the cyclin dependent kinase activating kinase associated with TFIIH  [ **ERCC3** ]  complex in stimulating transcription is mediated in part by p44 XPD  [ **ERCC2** ]  interaction.  Ref: 10924514 J Biol Chem, 2000
- Their deficiencies result from phenotype specific mutations in either XPB  [ **ERCC3** ]  or XPD  [ **ERCC2** ] .  Ref: 11335038 Trends Genet, 2001
- These findings extend the conservation of the NER apparatus and the link between NER and basal transcription and suggest that hMMS19 exerts its function in repair and transcription by interacting with the XPB  [ **ERCC3** ]  and XPD  [ **ERCC2** ]  helicases.  Ref: 11071939 Nucleic Acids Res, 2000
- Mutations in the XPD  [ **ERCC2** ]  helicase component of TFIIH  [ **ERCC3** ]  can result in the diverse clinical features associated with xeroderma pigmentosum XP and trichothiodystrophy TTD.  Ref: 11734544 Hum Mol Genet, 2001
- Three polypeptides, primarily positioned 5 to the damage, are in close physical proximity to the psoralen lesion and thus are cross linked to the damaged DNA these proteins are RPA70, RPA32, and the XPD  [ **ERCC2** ]  subunit of TFIIH  [ **ERCC3** ] .  Ref: 12138203 Mol Cell Biol, 2002
- This is an expected phenotype if the XPD  [ **ERCC2** ]  function is affected and indicates that the antisense approach may be an alternative in the study of TFIIH  [ **ERCC3** ]  functions in Drosophila.  Ref: 11334141 Antisense Nucleic Acid Drug Dev, 2001
- We conclude that the severity of the clinical features in TTD patients and the clinical outcome of differentially mutated XPD  [ **ERCC2** ]  proteins is likely to depend both on the effects that each mutation has on the stability of TFIIH  [ **ERCC3** ]  and on the transcriptional activity of the residual TFIIH  [ **ERCC3** ]  complexes.  Ref: 12393803 Hum Mol Genet, 2002
- Several NER genes in this pathway including XPB  [ **ERCC3** ] , XPD  [ **ERCC2** ] , XPA and ERCC 1 have been implicated in anticancer drug resistance in human tumor cells.  Ref: 12045463 Anticancer Drugs, 2002
- The antibody study indicated that they thus altered the conformation of one side of TFIIH  [ **ERCC3** ] , consisting of p44, XPD  [ **ERCC2** ] , and Cdk activating kinase subunits, that is essential for the transition stage.  Ref: 12665589 Mol Cell Biol, 2003
- Inherited mutations of the TFIIH  [ **ERCC3** ]  helicase subunits xeroderma pigmentosum XP B or XPD  [ **ERCC2** ]  yield overlapping DNA repair and transcription syndromes.  Ref: 11239393 Cell, 2001
- Several NER genes in this system including XPA, XPB  [ **ERCC3** ] , ERCC1, and  **ERCC2**  XPD  [ **ERCC2** ]  have been implicated in anticancer drug resistance in human tumor cells.  Ref: 12451985 Ai Zheng, 2002
- RESULTS Mammalian cell lines XPB  [ **ERCC3** ] , XPD  [ **ERCC2** ] , XPF, XPG, and ERCC1 deficient in the nucleotide excision repair NER gene products show resistance to Et 743.  Ref: 11731295 Chem Biol, 2001
- RESULTS The ERCC1, XPB  [ **ERCC3** ] , and XPD  [ **ERCC2** ]  protein expression patterns yielded significant negative Pearson correlations with 1 7, and 32 out of the 170 compounds, respectively P <.  Ref: 12451985 Ai Zheng, 2002
- The ERCC 1, XPD  [ **ERCC2** ]  and XPB  [ **ERCC3** ]  protein expression patterns yielded significant negative Pearson correlations with 1 2 and 17 out of the 170 compounds, respectively using p< ; 0.05.  Ref: 12045463 Anticancer Drugs, 2002
- In order to see if ERCC1 or XPD  [ **ERCC2** ]  activity was limiting for DNA repair, we cotransfected with plasmids encoding NER genes, thus over expressing either XPB  [ **ERCC3** ] , XPC, XPD  [ **ERCC2** ] , CSB or ERCC1 in the host cell reactivation assay.  Ref: 11056291 Mutat Res, 2000
- Mutations in several DExH containing DNA helicases, including XPD  [ **ERCC2** ] , XPB  [ **ERCC3** ] , WRN, and BLM, are associated with rare familial cancer syndromes characterized by genomic instability and cancer susceptibility.  Ref: 11765063 Acta Oncol, 2001
- We have also measured TFIIH  [ **ERCC3** ]  levels in cells in which different mutations in the XPD  [ **ERCC2** ]  gene are associated with clinical symptoms not of TTD but of the highly cancer prone disorder xeroderma pigmentosum XP.  Ref: 12393803 Hum Mol Genet, 2002
- We have recently completed screening of the National Cancer Institute human tumor cell line panel and demonstrated that among four nucleotide excision repair proteins XPA, XPB  [ **ERCC3** ] , XPD  [ **ERCC2** ] , and ERCC1, only the TFIIH  [ **ERCC3** ]  subunit XPD  [ **ERCC2** ]  endogenous protein levels correlate with alkylating agent drug resistance.  Ref: 12359753 Cancer Res, 2002
- The xeroderma pigmentosum group D XPD  [ **ERCC2** ]  helicase subunit of TFIIH  [ **ERCC3** ]  functions in DNA repair and transcription initiation.  Ref: 11242112 Nat Genet, 2001
- However, cells deficient in the other NER genes, XPD  [ **ERCC2** ] , XPB  [ **ERCC3** ]  and XPG, all have the same ratio of exchanges to breaks as wild type.  Ref: 12509251 DNA Repair Amst , 2002
- Regulation of cisplatin resistance and homologous recombinational repair by the TFIIH  [ **ERCC3** ]  subunit XPD  [ **ERCC2** ] .  Ref: 12359753 Cancer Res, 2002
- Here we show that the Drosophila TFIIH  [ **ERCC3** ]  component Xpd  [ **ERCC2** ]  negatively regulates the cell cycle function of Cdk7, the CAK activity.  Ref: 12853965 Nature, 2003
- In this report, we demonstrate an association of a C terminal domain of human RAD52 amino acids 302 418 with the XPB  [ **ERCC3** ]  and XPD  [ **ERCC2** ]  subunits of transcription factor TFIIH  [ **ERCC3** ]  and RNA polymerase II RNAPII.  Ref: 12372413 Biochem Biophys Res Commun, 2002
- Mutations in XPB  [ **ERCC3** ]  and XPD  [ **ERCC2** ]  TFIIH  [ **ERCC3** ]  helicases have been related with three hereditary human disorders xeroderma pigmentosum, Cockayne syndrome, and trichothiodystrophy.  Ref: 12221129 Mol Biol Cell, 2002
- The XPD  [ **ERCC2** ]  protein performs not only an essential viability function as a structural component of transcription initiation factor TFIIH  [ **ERCC3** ] , but also an NER function as a 5 to 3 DNA helicase within TFIIH  [ **ERCC3** ]  that unwinds DNA on the 3 side of bulky lesions.  Ref: 11746743 Environ Mol Mutagen, 2001
- Loss of heterozygosity LOH analysis, using the microdissected tissues, for the XPA, XPB  [ **ERCC3** ] , XPC, XPD  [ **ERCC2** ] , XPE, XPF, XPG and the transcription coupled repair factor, Cockayne syndrome B CSB revealed that NER factors were abnormal in 30.0% 3 10 cases of oral squamous cell carcinomas.  Ref: 11496330 Oncol Rep< MedlineTA>Oncol Rep, 2001
- Co immunoprecipitation experiments revealed that hMMS19 directly interacts with the XPB  [ **ERCC3** ]  and XPD  [ **ERCC2** ]  subunits of NER transcription factor TFIIH  [ **ERCC3** ] .  Ref: 11071939 Nucleic Acids Res, 2000
- We report here the different ways in which four subunits of the basal transcription repair factor TFIIH  [ **ERCC3** ]  XPB  [ **ERCC3** ] , XPD  [ **ERCC2** ] , p62 and p44 and the damage recognition XPC repair protein can enter the nucleus.  Ref: 11266560 Nucleic Acids Res, 2001
- It is not surprising that under these conditions some XPD  [ **ERCC2** ]  mutations affect interactions not only with p44, but also with MAT1, thus preventing either the CAK inhibitory function within CAK.XPD  [ **ERCC2** ]  and or the role of CAK within TFIIH  [ **ERCC3** ]  and, consequently, explaining the variety of the XP phenotypes.  Ref: 11445587 J Biol Chem, 2001
- Herein we report that HBx represses two components of the transcription repair factor TFIIH  [ **ERCC3** ] , XPB  [ **ERCC3** ]  p89, and XPD  [ **ERCC2** ]  p80, both in p53 proficient and p53 deficient liver cells.  Ref: 11278765 J Biol Chem, 2001
- Inherited mutations in the XPD  [ **ERCC2** ]  subunit of the general transcription repair factor TFIIH  [ **ERCC3** ]  yield the rare genetic disorder Xeroderma pigmentosum XP, the phenotypes of which cannot be explained solely on the basis of a DNA repair defect.  Ref: 11955452 Cell, 2002
- Expression of HBx in liver cells results in down regulation of endogenous XPB  [ **ERCC3** ]  and XPD  [ **ERCC2** ]  mRNAs and proteins.  Ref: 11278765 J Biol Chem, 2001
- We have shown previously that mutations in the C terminus of the XPD  [ **ERCC2** ]  helicase, another subunit of TFIIH  [ **ERCC3** ] , prevent its regulation by p44 Coin, F.  Ref: 10924514 J Biol Chem, 2000
- Such proteins include BRCA1, ATM, WRN, BLM, p53, XPB  [ **ERCC3** ] , XPD  [ **ERCC2** ] , MSH2, MSH6, MLH1 and PMS2.  Ref: 12052432 Mutat Res, 2002
- In the Sp1 deficient SL2 cells, HBx induced XPB  [ **ERCC3** ]  and XPD  [ **ERCC2** ]  inhibition is Sp1 dependent.  Ref: 11278765 J Biol Chem, 2001
- Because XPB  [ **ERCC3** ]  and XPD  [ **ERCC2** ]  function in both nucleotide excision repair and transcription initiation, the cellular phenotypes associated with a deficiency of each one of them include failure to repair mutagenic DNA lesions and defects in the recovery of RNA transcription after UV irradiation.  Ref: 11701636 Annu Rev Genomics Hum Genet, 2000
- Within the ring structure, p44 was flanked on either side by the XPB  [ **ERCC3** ]  and XPD  [ **ERCC2** ]  helicases.  Ref: 11007478 Cell, 2000
- Here, XPB  [ **ERCC3** ]  and XPD  [ **ERCC2** ]  mutations are shown to block transcription activation by the FUSE Binding Protein FBP, a regulator of c myc expression, and repression by the FBP Interacting Repressor FIR.  Ref: 11239393 Cell, 2001
- mRNA levels of XPA, XPB  [ **ERCC3** ] , XPC, XPD  [ **ERCC2** ] , XPF, XPG, CSB and ERCC1 in primary lymphocytes from 33 individuals were quantified by dot blots and normalized to beta actin.  Ref: 11056291 Mutat Res, 2000
- Another class of patients with CS symptoms have mutations in the XPB  [ **ERCC3** ] , XPD  [ **ERCC2** ] , or XPG genes, which result in UV hypersensitivity as well as defective global NER.  Ref: 11443545 Am J Hum Genet, 2001
- Loss of heterozygosity LOH analysis for the XP, XPA, XPB  [ **ERCC3** ] , XPC, XPD  [ **ERCC2** ] , XPE, XPF, XPG and the transcription coupled repair factor, Cockayne syndrome B CSB revealed that NER factors were abnormal in 62.1 % of ovarian tumors 18 2 6.7% of colon 2 12 and 22.2% lung 2 9 carcinomas.  Ref: 11689286 Cancer Lett, 2001
- Mutations in XPB  [ **ERCC3** ]  and XPD  [ **ERCC2** ]  can result in xeroderma pigmentosum, Cockayne syndrome, or trichothiodystrophy.  Ref: 11701636 Annu Rev Genomics Hum Genet, 2000
- In liver tissue from HBx transgenics, XPB  [ **ERCC3** ]  and XPD  [ **ERCC2** ]  proteins are down regulated in comparison to matched normal liver tissue.  Ref: 11278765 J Biol Chem, 2001
